# Green iron oxide nanoparticles and magnetic nanobiochar: enhancing tomato performance, phytochemicals, and root-knot nematode resistance

**DOI:** 10.1186/s12870-024-05131-3

**Published:** 2024-05-29

**Authors:** Nashaat N. Mahmoud, Asmaa Khader, Esawy Mahmoud

**Affiliations:** 1 Department of Botany and Microbiology, Faculty of Science, Al-Azhar University, Nasr city, Egypt; 2 Water and Environment Research Institute, Agricultural Research Center, Sakha, Egypt; 3 Soil and Water Department, Faculty of Agriculture, Tanta University, Tanta, Egypt

**Keywords:** Green nanoparticles, Root-knot nematodes, Phytochemicals, Surface area, Green magnetic nanobiochar, Tomato growth

## Abstract

**Background:**

Green nanoparticles are considered to be an effective strategy for improving phytochemicals and raising productivity in soil infected by root-knot nematodes. This work aims to understand the characteristics of certain nanomaterials, including non-iron (nFe), green non-iron (GnFe), and green magnetic nanobiochar (GMnB), and the effect of adding them at 3 and 6 mg kg^− 1^ on phytochemicals and tomato (*Solanum lycopersicum)* plant growth in soils infected by root-knot nematodes.

**Results:**

Spectroscopic characterization of nanomaterials showed that nFe, GnFe, and GMnB contained functional groups (e.g., Fe-O, S-H, C-H, OH, and C = C) and possessed a large surface area. Application of GMB at 6 mg kg^− 1^ was the most efficient treatment for increasing the phytochemicals of the tomato plant, with a rise of 123.2% in total phenolic, 194.7% in total flavonoids, 89.7% in total carbohydrate, 185.2% in total free amino acids, and 165.1% in total tannin compared to the untreated soil. Tomato plant growth and attributes increased with increasing levels of soil nano-amendment in this investigation. The addition of GnFe_3_ and GnFe_6_ increased the reduction of root galls of root-knot nematodes by 22.44% and 17.76% compared with nFe_3_ and nFe_6_, respectively. The inclusion of the examined soil nano-amendments increased phytochemicals and reduced the total number of root-knot nematodes on tomato plants at varying rates, which played a significant role in enhancing tomato growth.

**Conclusions:**

In conclusion, treating tomato plants with GnFe or GMnB can be used as a promising green nanomaterial to eliminate root-knot nematodes and increase tomato yield in sandy clay loam soil.

## Background

Egypt ranks fifth in the production of tomatoes, scheduled to come in first behind China, India, Turkey, and the United States of America, with a cultivable area of 170,862 ha and an output of 6,731,220 tons in 2020 [1]. Commonly known as the edible berry, the tomato (*Solanum lycopersicum*) belongs to the Solanaceae family. It is considered one of the most important vegetable plants in the world, and it originated in western South America and Central America [2]. Tomatoes are high in nutrients and bioactive components such as carotenoids, ascorbic acid, vitamin E, and phenolic compounds, which provide antioxidant activity by neutralizing reactive oxygen species (ROS) and protecting the cell membrane from lipid peroxidation. It can aid in the treatment of various disorders, particularly chronic conditions [3]. Tomatoes are recognized for having a variety of flavonoids along with phenolics that can aid in the prevention and treatment of inflammation, coronary artery disease, and cancer, as well as a balanced diet [3, 4].

Tomato plants are infected with the root-knot nematode *Meloidogyne incognita*, causing significant losses in yield and nutritional value in both greenhouses and field studies [5]. Root-knot nematode species, *M. incognita* and *M. javanica*, are major species distributed worldwide and parasitize a wide range of economic crops. Jones et al. [6] showed that root-knot nematodes rank first among the 10 most dangerous plant pathogens, causing economic losses estimated at $77–80 billion annually globally [7]. Most tomato plant losses are caused by plant parasitic nematodes (PPN) genera, those that feed on roots and aerial parts, such as root-knot nematodes (*Meloidogyne* spp.) and *cys*t nematodes. In response to pathogenic infections, tomato plants showcase a spectrum of reactions, from acoustic cues to physiological shifts and phytochemical adaptations [8]. Plant-parasitic nematode control is primarily based on synthetic organic chemical nematicides [9]. Because of the expensive expense of these compounds and their environmental impact, scientists have turned to other methods of combating nematodes and green nematicidal products that are less harmful and environmentally benign while also working to boost yields [10]. So, the green synthesis of nanoparticles is the preferred technique for treating plant diseases because of its low levels of toxicity, low production cost, and ability to improve plant growth [11–14]. Green hematite nanoparticles improved the uptake of nutrients, sorghum growth, and osmoregulation in drought-stressed plants by reducing oxidative damage to biomolecules [15]. Iron is a critical component of cellular redox processes, acting as a precursor for anti-oxidative enzymes like catalase (CAT), superoxide dismutase (SOD), and peroxidase (POD), as well as a scavenger of ROS [16]. An excessive amount of ROS is produced by plants when they are exposed to abiotic stress, either alone or together, resulting in oxidative stress and disturbed redox balance [17, 18]. Aside from their adverse effects, ROS play an important role as secondary messengers or signaling molecules in a variety of cellular mechanisms that boost tolerance to various abiotic stresses [19], particularly during adaptation processes. The harmony between ROS production and antioxidant resistance keeps plants against the effects of stress [18, 20]. And also, as a result, by producing secondary metabolites, these substances create the perfect environment for plant improvement and stress resistance [21]. There have been no studies on the use of green nanomaterials in eliminating root-knot nematodes and producing secondary metabolites so far. Therefore, the novelty of this research is the use of green iron oxide nanoparticles and magnetic nanobiochar in agriculture to reduce root-knot nematode disease and increase tomato productivity.

It is hypothesized in the present study that the addition of nFe alone will not be able to completely reduce root-knot nematode disease in infected tomato plants and the production of phytochemicals. Therefore, the study’s goal was to investigate the effects of GnFe or GMnB application at various rates on phytochemicals and root-knot nematodes of tomato plants, as well as tomato performance.

## Materials and methods

### Studied area

The soil tests were gathered in the vicinity of El Nubaria City in Egypt’s Beheira Governorate. The area is located at 30° 9’ 11.52” N and 30° 40’ 59.88” E. According to Abu El Enain et al. [22], the soil is classified as *Haplocalcid* (Aridisols order). To create the composite sample used in the experiment, disturbed samples (about 10 samples from different farms in the same area) are collected at a depth of 0–20 cm and mixed together properly to obtain a homogeneous mixture. The characteristics of the examined soil are presented in Table 1.

**Table 1:** Properties of soil and nano-amendments used in this study

Properties	units	Soil	CT	nFe	GnFe	GMnB
pH		7.79	8.01	4.2	6.1	7.03
EC	dSm^-1^	1.85	4.08	0.01	0.92	2.44
OC	%	0.62	24.6	-	-	48.03
CEC	cmol kg^-1^	29.03	-	-	-	35.7
Total Al	%	-	-	0.25	-	-
Available P	mg kg^-1^	16.02	-	-	-	-
Available K		230	-	-	-	--
Available N		21	5600	-	-	-
Total N	%	-	0.42	-	-	4.07
Total P		-	0.36	-	-	4.54
Total K		-	0.49	-	-	5.93
Texture		Sandy clay loam	-	-	-	-

Where, CT: Compost tea; nFe: Nano iron oxide; GnFe; Green nano iron oxide; GMnB: Green magnetic nanobiochar; CEC: Cation exchange capacity; EC: Electrical conductivity; OC: Organic carbon

### Synthesis iron nanoparticles (nFe)

2.5 M NaOH solution was added dropwise to 200 ml of 0.0651 M iron metal salt (FeSO_4_.7H_2_O) solution up to pH 11. The mixture is then heated at 170 °C for 75 min to precipitate the magnetic (black) iron oxide. The solid nanoparticles (black precipitate) were then obtained by vacuum filtering the mixture. Finally, the solid nanoparticles were washed three times with distilled water and ethanol to remove the remaining salt solution from the sample’s surface. The magnetite obtained is dried overnight in a fume hood, stored in a desiccator, and crushed using a mortal pestle [23].

### Synthesis of green iron nanoparticles (GnFe)

Compost tea (rice straw as raw material and use of ground fertilizer) is rich in secondary metabolites that act as capping and reducing agents, which were taken from the Microbiology Unit, Microbiology Department, Agricultural Research Center, Egypt, and used in the production of green iron. Nanoparticles (GnFe).

Compost tea (using rice straw as feedstock and turned windrow composting) is rich in secondary metabolites that act as capping and reducing agents. This was taken from the Microbiology Unit, Department of Microbiology, Agricultural Research Center, Egypt, and used to create green iron nanoparticles (GnFe). In a 500-ml beaker, combine 7.0 ml of a 2 mM FeSO_4_.7H_2_O solution plus 10 ml of compost tea, stir for 5 min at 25^o^C, and set the pH to more than 8. Within 5 min, the color of the mixture altered from translucent yellow to black, suggesting nFe synthesis. The mixture was subsequently separated by a vacuum filter to yield solid nanoparticles that were washed three times using ethanol and distilled water, respectively, and then centrifuged at 1000 rpm for 3 min and dried at 35 °C for 24 h.

### Synthesis of green magnetic nanobiochar (GMnB)

1.2 g of magnetic nanobiochar MnB (magnetic nanobiochar has been previously synthesized by Khader et al. [24]) was combined with 200 mL of compost tea for 0.5 h at 25 °C. The resulting mixture was subsequently vacuum-filtered to isolate the solid nanoparticles, which were then washed three times using ethanol and distilled water, respectively, and then centrifuged at 1000 rpm for a total of three minutes before being air-dried at 35 °C for 24 h.

### Extraction of nematode eggs

RKN inoculum was extracted for the investigations by harvesting infected tomato plants. The galled roots were carried to the lab and cleaned with water to eliminate any dust that had accumulated. The roots were cut into 0.5–3 cm pieces with sterile scissors and agitated for 2–3 min in a 1% sodium hypochlorite solution before being passed through a succession of sieves of varying sizes (150, 250, and 350 mm). They were subsequently added to a 100-mL beaker containing 50 mL of sterile distilled water until use [25]. Peters’ chamber determined the quantity of eggs and modified it to 100 eggs per mL.

### Pot experiment

**Fig. 1:**
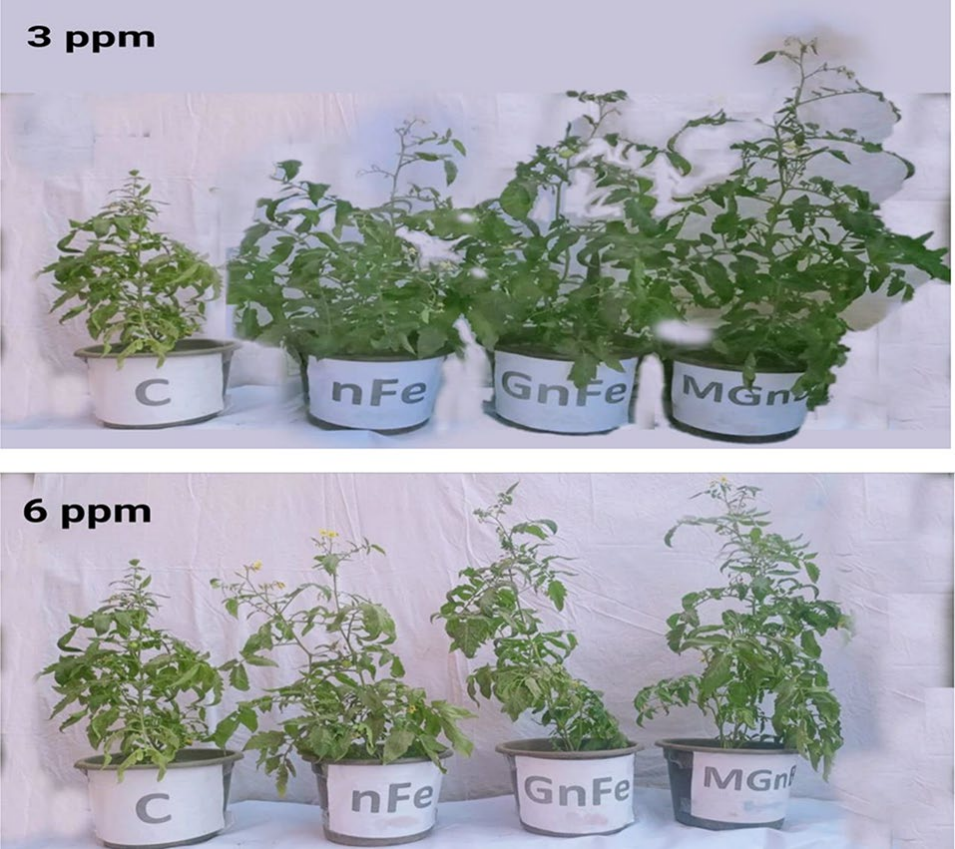
Experiment pots of tomato plants after 70 days of seedling. Where C: Control; nFe : Iron nanoparticles; GnFe : Green iron nanoparticles; and GMnB: Green magnetic nanobiochar at a rate of 3 and 6 g kg^-1^

Between July 1st and September 5th, an open-air pot trial was carried out in Basuon village (30^o^ 57` N, 30^o^ 49` E) in Gharbia Governorate, Egypt. Seven treatments were conducted in a randomized complete trial design with five replications, as follows: C: control (NPK recommended doses); nFe_3_: nano iron oxide at 3 mg kg^− 1^; nFe_6_: nano iron oxide at 6 mg kg^−^; GnFe_3_: green nano iron oxide at 3 mg kg^− 1^; GnFe_6_: green nano iron oxide at 6 mg kg^− 1^; GMnB_3_: green magnetic nanobiochar at 3 mg kg^− 1^, and GMnB_6_: green magnetic nanobiochar at 6 mg kg^− 1^. In this study, the rates were based on a pervious study by Zhou et al. [26]. After 40 days, tomato (*Solanum lycopersicum*) seedlings of variety 186 were transferred to plastic pots (30 cm in diameter and 25 cm in height) containing 10 kg of composite soil and irrigated based on water requirements (based on 75% of the field capacity). A suspension of nanomaterials (nFe, GnFe, and GMnB) was made in 250 mL of water, dipped tomato seedlings in it, then planted in the soil and added the rest of the suspension next to the root area. Three weeks after planting the seedlings, the tomato seedlings were infected with root-knot nematodes by inserting about 5,000 eggs by making six holes in the ground near the root area using a glass rod to avoid infecting the roots of the tomato seedlings [18]. NPK fertilization rates for tomatoes are advised by the Egyptian Ministry of Agriculture. Before cultivation, phosphorus was given at a rate of 142 kg ha^− 1^ as superphosphate (15.5% P_2_O_5_). Three times as much nitrogen was given in equal doses at a rate of 285 kg ha^− 1^ as urea (46%). At a rate of 220 kg ha^− 1^, potassium was added as potassium sulfate (48% K_2_O). All other agricultural practices in the experiment were done as recommended in the study area. During the experiment periods, the temperature ranged from 16 °C to 29 °C, the relative humidity ranged from 45 to 60%, and there was no rainfall. Plants were collected 70 days after seedling emergence (Fig. 1), and nematode parameters, phytochemical analysis, and plant growth characteristics were assessed.

### Plant growth characteristics

Following nine weeks of sowing, measurements of different plant growth characteristics, such as root length, shoot length, fresh weight, and dry weight, were made. From the initial flower’s appearance to the sixth week, the total number of flowers was counted [27].

### Total flavonoid concentrations

According to Zhishen et al. [28], the total flavonoid concentrations were determined using an aluminum chloride (AlCl_3_) colorimetric technique. 1 mL of fresh plant extract was combined with 0.3 mL of 5% NaNO_2_. After a period of minutes, a total of 2 mL of 1 M NaOH and 0.3 mLof AlCl_3_ were introduced. After allowing the chemical reaction combination to stand for several minutes, the wavelength of absorption at 510 nm was evaluated versus a blank solution of the reaction. The flavonoid concentrations in these extracts were calculated as mg quercetin equivalents per fresh weight, employing a quercetin curve as a standard.

### Total phenol content

Plant extract (1 mL) was combined with Folin-Ciocalteu’s phenol solution (1 ml mixed 1:10 with distilled water), and then 2 ml of Na_2_ CO_3_ (7.5% w/v) was added. Following that, the test tubes were wrapped in aluminum foil, shaken, and incubated for two hours. The absorbance at the wavelength of 765 nm was measured as a blue color, as indicated for phenolic substances. The total phenol content was reported in mg gallic acid equivalents per g of extract [29].

### Total amino acids and tannins content of tomato plants

The total free amino acid concentration has been determined using the Hamilton et al. [30] method. 1 mL of plant extract was combined with 1 mL of pyridine solution (10%) and 1 ml of ninhydrin solution (2%), and the mixture was left at ambient temperature for 30 min. At 570 nm, the absorbance of the resulting solution was measured.

The tannin content of tomato plants was measured according to the Folin-Denis method [31]. Saponin content was determined according to Okwu and Ukanwa [32].

### Analysis of plant and soil samples

The dry weight of the plant samples was weighed after drying them in the oven for forty-eight hours at 70 °C. Soil physicochemical properties were determined using the procedures described by Cottenie et al. [33] and Page et al. [34].

Specific surface area (SSA) was calculated by the Sauter formula: S = 6000/ρ × D.

Where S is the specific surface area, ρ is the density of the synthesized material, and D is the size of the particles [35].

### Spectroscopic analysis

#### TEM nanoparticle analysis

Transmission electron microscopy (TEM) was used to measure the particle size of different types of iron oxide nanoparticles and their morphology, which was performed using a microscope type FEI TECNAI G20 (200 KV-LaB6 emitter). Fourier transform infrared spectroscopy (FTIR) was used to determine the functional groups on the nano-amendments. FTIR was used with TENSOR 27 by Bruker, which was prepared using KBr as a sample medium, to confirm the results with an analysis in the range 400–4000 cm^− 1^. X-ray diffraction (XRD) was used to determine the crystallographic structure of the studied nanomaterials. The samples were recorded from 15^◦^ to 75^◦^ and tested using a GNR X-ray Diffractometer (Model: APD 2000 PRO).

### Nematode parameters

Using the Bridge and Page grading scale, the quantity of galls on the roots of each treatment was assigned a rating [36]. The amount of egg mass generated from root systems was also evaluated [37]. Each pot’s 250 cm^3^ of soil was subjected to a 48-hour separating time and sifting (250, 350 m) in order to extract the nematodes [38]. After releasing the eggs from each root system with 1% sodium hypochlorite, the total number of eggs was counted. The number of eggs suspended in water was then counted using a stereoscopic microscope.

### Statistical analysis

All obtained data were analyzed statistically using DSAASTAT version 1.101 software; five replicas were utilized for the statistical evaluation of variance (ANOVA). The Duncan’s Multiple Range Test (DMRT) was used to compare treatments with a statistical significance level of *P* < 0.05. The relationship between quantitative statistical data was represented by the correlation, which was computed using Microsoft Office 365.

## Results

### Spectroscopic analyses of the studied nanomaterials

**Fig. 2:**
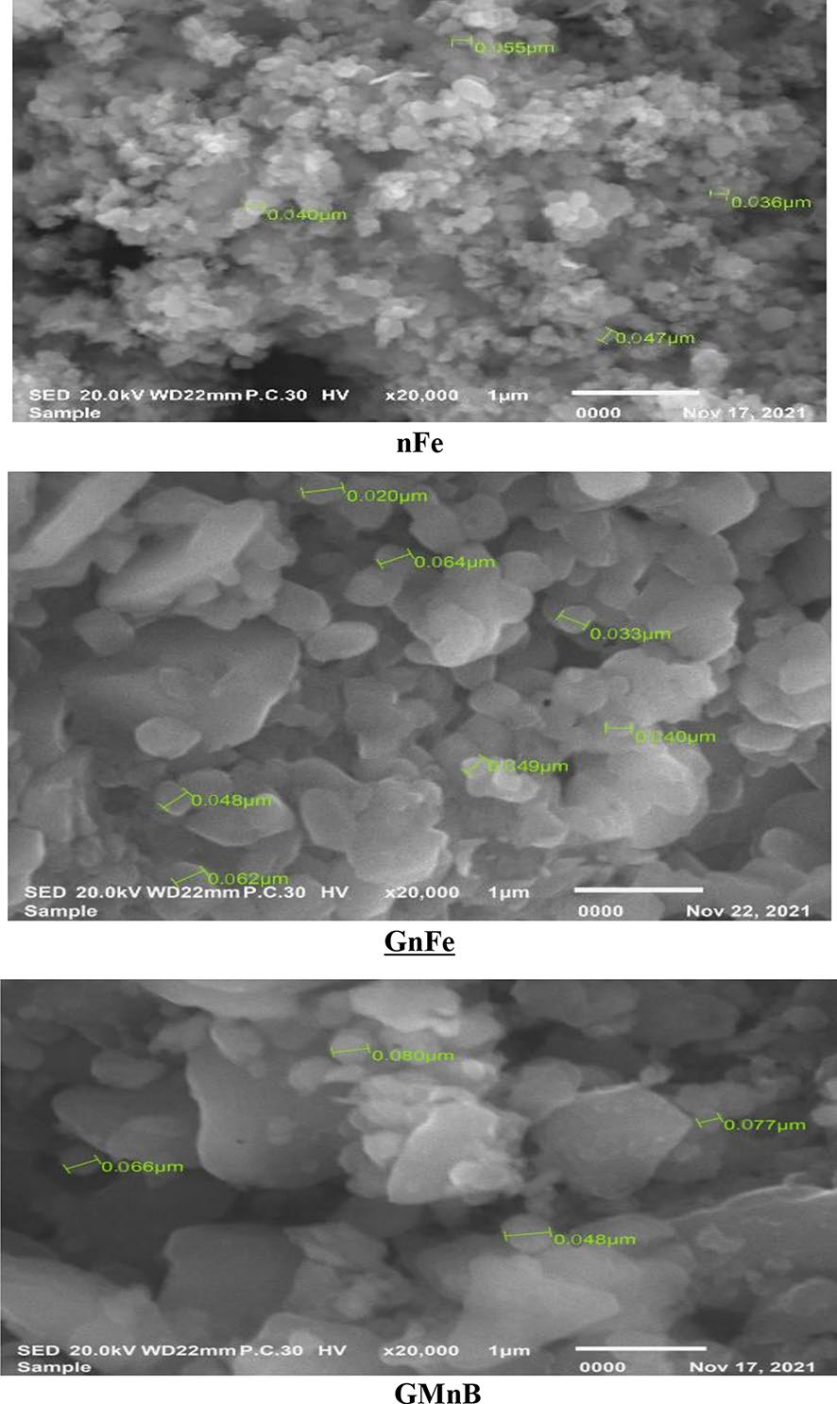
Transmission electron microscopy (TEM) images of particle size measurements of iron nanoparticles (nFe), green iron nanoparticles (GnFe), and green magnetic nanobiochar (GMnB)

TEM images of the synthesized morphologies of nFe, GnFe, and GMnB are presented in Fig. 2. It is noted that the surfaces of nFe are spherical with a moderate difference in size. The particles agglomerate with each other in a size range of 36 to 55 nm. While GMnB was characterized by a cover on the surface particles, the presence of some pores, and irregular shapes of different sizes, the particles agglomerated with each other with a size range of 48 to 80 nm. TEM analysis of GnFe showed that the shape of the nanoparticles is rod, rhomboid, and other irregular shapes, and they agglomerate with each other in the form of flower clusters with a size range of 33 to 64 nm. The surface area of GMnB was 304.38 m^2^ g^− 1^, which is larger than that of nFe (92.10 m^2^ g^− 1^) and GnFe (190.9 m^2^ g^− 1^).

**Fig. 3:**
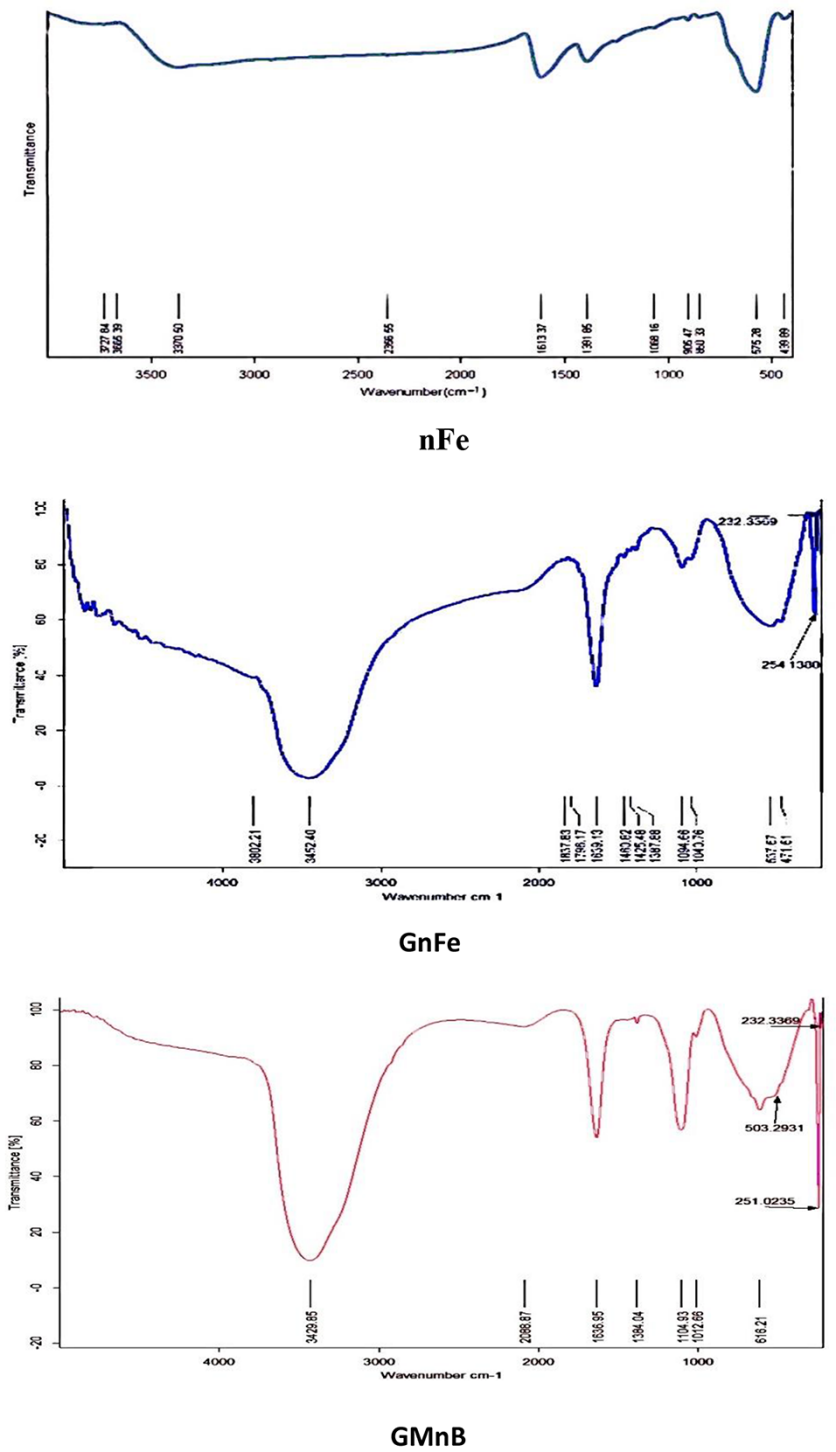
Fourier transform infrared spectroscopy (FTIR) identifies functional groups on iron nanoparticles (nFe), green iron nanoparticles (GnFe), and green magnetic nanobiochar (GMnB)

Figure 3 shows the FTIR of the studied nanomaterials containing many peaks, such as peaks at 3665.39 cm^− 1^, 3370.50 cm^− 1^, 2366.55 cm^− 1^, 1613.37 cm^− 1^, 1391.83 cm^− 1^, 1088.16 cm^− 1^, 860.33 cm^− 1^, and 575.38 cm^− 1^ in nFe, 3452.40 cm^− 1^, 3802.21 cm^− 1^, 2088.87 cm^− 1^, 1460.82 cm^− 1^, 537.67 cm^− 1^, and 503 cm^− 1^ in GnFe, and 3429.85 cm^− 1^, 3429.85 cm^− 1^, 1639.13 cm^− 1^, 1104 cm^− 1^, 60 2 cm^− 1^, and 575.28 cm^− 1^ in GMnB.

**Fig. 4:**
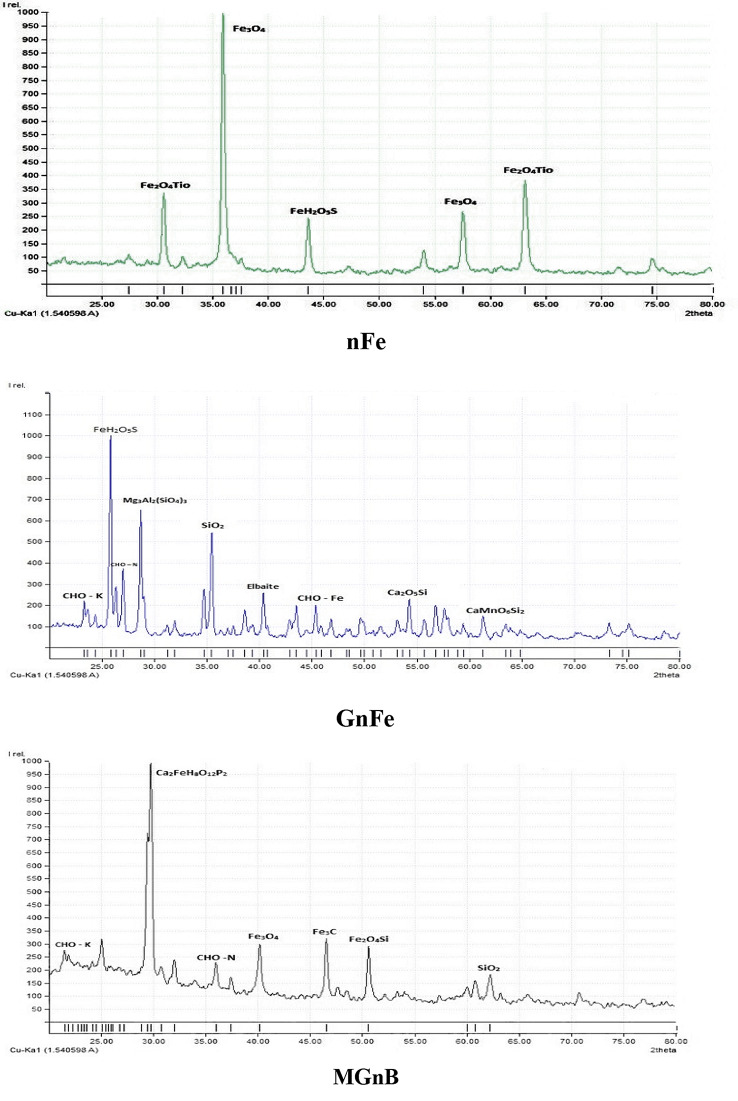
X-ray diffraction (XRD) spectrum for iron nanoparticles (nFe), green iron nanoparticles (GnFe), and green magnetic nanobiochar (GMnB)

The X-ray diffraction (XRD) patterns of the three amendments (nFe, GnFe, and GMnB) are shown in Fig. 4. The results show the spinel phase structure of magnetite (Fe_3_O_4_), Fe_3_O_4_TiO, and ferrous sulfate monohydrate in the nFe. The peaks at 2ϴ = 24.2°, 25.0°, 27.4°, 35.0°, 45.0°, and 46.8° were identified for GnFe and GMnB. GnFe also contains many minerals, such as elbaite, pyrope Ca_3_Al_2_ (SiO_4_), and johannesita. While the peaks at 2ϴ = 29.5°, 40.0°, 47.8°, 51.0°, and 62.5° in GMnB were identified as anapait (Ca_2_;Fe²^+^(PO_4_;)_2_;·4 H_2_;O.), iron oxide (Fe_2_O_2_), iron carbide (Fe_3_C), asimowite (Fe_2_O_4_Si), and quartz (SiO_2_), respectively.

### Effect of nano-amendments on of phytochemicals of tomato plant

Table 2 showed that non-enzymatic antioxidant compounds such as phenols, flavonoids, free amino acids, total carbohydrate, and tannins in nematode-infected tomato plants were significantly affected with the addition of nFe, GnFe, and GMnB at different concentrations. Total phenolic content (TPH) in tomato plants varied from 10.42 mg GA g^− 1^ DW in the control to 23.25 mg GA g^− 1^ DW in the GMnB_6_. The TPH contents rose as the application rates of the assessed nanoparticles increased. TPH rose by 36.85% and 23.34%, respectively, with the integration of GnFe_3_ and GnFe_6_, in contrast to the supplementary addition of nFe_3_ and nFe_6_, respectively. The addition of GMnB_3_ and GMnB_6_ boosted TPH by 1.61 and 2.23 times, respectively, compared to the untreated soil. At the same rate, the TPH in the GMnB-treated pots was greater than in the MnB-treated pots. Total flavonoids ranged from 12.93 mg Rutin g^− 1^ DW in the control to 38.1 mg Rutin g^− 1^ DW in the GMnB_6_ treatment. There was a noticeable difference between the various treatments in the total proteins (TP) of tomato plants, which ranged from 13.71 g BSA 100 g^− 1^ DW in the control to 26.46 g BSA 100 g^− 1^ DW in the GMnB_6_ treatment. When compared to the addition of nFe_3_ and nFe_6_, respectively, the addition of GnFe_3_ and GnFe_6_ enhanced TP by 25.15% and 19.49%, respectively. With increasing rates of soil nano-amendments introduced, tomato plant TP increased. The total carbohydrate, total free amino acid, and total tannin contents of tomato plants significantly increased in the pots treated with the nFe, GnFe, and GMnB additions. Total carbohydrate content was found to be higher in the GMnB_6_ treatment (62.04 g glucose 100 g^− 1^ DW) and lower in the control (32.71 g glucose 100 g^− 1^ DW). Total tannins in tomato plants rose from 6.67 to 11.67 mg TA g^− 1^ DW and from 6.67 to 17.68 mg TA g^− 1^ DW in soil treated with GMnB at 3 mg kg^− 1^ and 6 mg kg^− 1^ addition rates, respectively. In this investigation, total carbohydrate, total free amino acid, and total tannin contents increased with increasing levels of soil nano-amendment. There was no statistically significant difference in total carbohydrate, total free amino acid, or total tannin content among the nFe and GnFe treatments at 3 mg kg^− 1^. Total tannins in tomato plants rose from 6.67 to 11.67 mg TA g^− 1^ DW and from 6.67 to 17.68 mg TA g^− 1^ DW in soil treated with GMnB at 3 mg kg^− 1^ and 6 mg kg^− 1^ addition rates, respectively.

**Table 2:** Effect of the studied nanomaterials on of phytochemicals of tomato plant

Treatments	Total carbohydrates, g glucose 100 g^-1^ DW	Total proteins, g BSA 100 g^-1^ DW	Total free amino acids, g leucine 100 g^-1^ DW	Total flavonoids, mg Rutin g^-1^ DW	Total phenolic acids, mg GA g^-1^ DW	Total tannins, mg TA g^-1^ DW
C	32.71 ^e^	13.71 ^f^	4.26 ^e^	12.93 ^e^	10.42 ^e^	6.67 ^e^
nFe_3_	55.14 ^d^	23.39 ^e^	10.98 ^d^	28.27 ^d^	11.75 ^d^	8.24 ^d^
nFe_6_	55.58 ^c^	23.58 ^d^	11.05 ^c^	31.1 ^c^	16.75 ^b^	9.9 ^c^
GnFe_3_	55.14 ^d^	23.39 ^e^	10.98 ^d^	28.27 ^d^	16.08 ^c^	8.24 ^d^
GnFe_6_	55.58 ^c^	24.58 ^c^	11.05 ^c^	31.1 ^c^	16.75 ^b^	9.9 ^c^
GMnB_3_	59.04 ^b^	25.16 ^b^	11.78 ^b^	37.6 ^b^	16.75 ^b^	11.67 ^b^
GMnB_6_	62.04 ^a^	26.46 ^a^	12.15 ^a^	38.1 ^a^	23.25 ^a^	17.68 ^a^
F	**	**	**	**	**	**
LSD _0.05_	1.75	0.0175	1.75	1.75	1.75	1.75

Where: C: control (NPK recommended doses); nFe_3_: nano iron oxide at 3 mg kg^-1^; nFe_6_ :nano iron oxide at 6 mg kg^-^; GnFe_3_; green nano iron oxide at 3 mg kg^-1^; GnFe_6_: green nano iron oxide at 6 mg kg^-1^; GMnB_3_: green magnetic nanobiochar at 3 mg kg^-1^, and GMnB_6_: green magnetic nanobiochar at 6 mg kg^-1^. *Note: values of each now followed by the same letter indicate no significant differences (*p* ≤ 0.05) according to Duncan test

### Effect of nano-amendments on tomato plant growth and attributes

All nano-amendments significantly improved tomato plant growth and attributes (Table 3). The addition of nFe_3_ and nFe_6_ boosted the dry weight of the plant by 1.51 and 1.72 times, respectively, compared to the untreated soil. Tomato plant growth and attributes increased with increasing levels of soil nano-amendment in this investigation. Application of GMnB at 6 mg kg^− 1^ was the most efficient treatment for increasing plant fresh weight, with a rise of 102.6% in dry weight and 40.2% in root length compared to the untreated soil. There was no statistically significant difference in root length, number of leaves, or fresh weight of the tomato plant among the GMB_3_ and GMB_6_ treatments.

**Table 3:** Effect of iron nanoparticles (nFe), green iron nanoparticles (GnFe), and green magnetic nanobiochar (GMnB) at various rates on tomato growth

Treatments	Root length, cm	Number of leaves	Number of flowers	Number of laterals	Fresh weight of plant, g	Dry weight of plant, g	Plant height, cm
C	20.60 ^**g**^	15.67 ^**g**^	12.00 ^**f**^	18.00 ^**g**^	14.68 ^**f**^	12.40 ^**f**^	12.40 ^**f**^
nFe_3_	27.07 ^**f**^	20.00 ^**f**^	19.00 ^**e**^	28.00 ^**f**^	76.76 ^**e**^	18.71 ^**e**^	75.67 ^**e**^
nFe_6_	27.67 ^**f**^	23.00 ^**d**^	22.00 ^**c**^	27.00 ^**f**^	76.37 ^**e**^	21.32 ^**d**^	80.00 ^**d**^
GnFe_3_	26.45 ^**c**^	28.00 ^**bc**^	22.62 ^**c**^	31.23 ^**b**^	81.78 ^**b**^	22.75^**c**^	83.35^**c**^
GnFe_6_	26.07 ^**b**^	28.31 ^**bc**^	24.15 ^**b**^	35.08 ^**b**^	84.63 ^**b**^	22.12^**c**^	88.60^**c**^
GMnB_3_	27.66 ^**a**^	31.77 ^**ab**^	26.50 ^**b**^	40.03 ^**a**^	85.19 ^**a**^	23.50^**b**^	90.30^**b**^
GMnB_6_	28.88 ^**a**^	32.48 ^**a**^	27.19 ^**a**^	41.04 ^**a**^	86.04 ^**a**^	25.12^**a**^	92.63 ^**a**^
F-test	**	**	**	**	**	**	**
LSD _(0.05)_	0.60	0.61	0.22	1.33	1.94	0.59	1.01

Where: C: control (NPK recommended doses); nFe_3_: nano iron oxide at 3 mg kg^-1^; nFe_6_ :nano iron oxide at 6 mg kg^-^; GnFe_3_; green nano iron oxide at 3 mg kg^-1^; GnFe_6_: green nano iron oxide at 6 mg kg^-1^; GMnB_3_: green magnetic nanobiochar at 3 mg kg^-1^, and GMnB_6_: green magnetic nanobiochar at 6 mg kg^-1^. Similar letters indicate no significant variations among treatments

### Effect of nanomaterials on tomato plant root-knot nematode populations

**Fig. 5:**
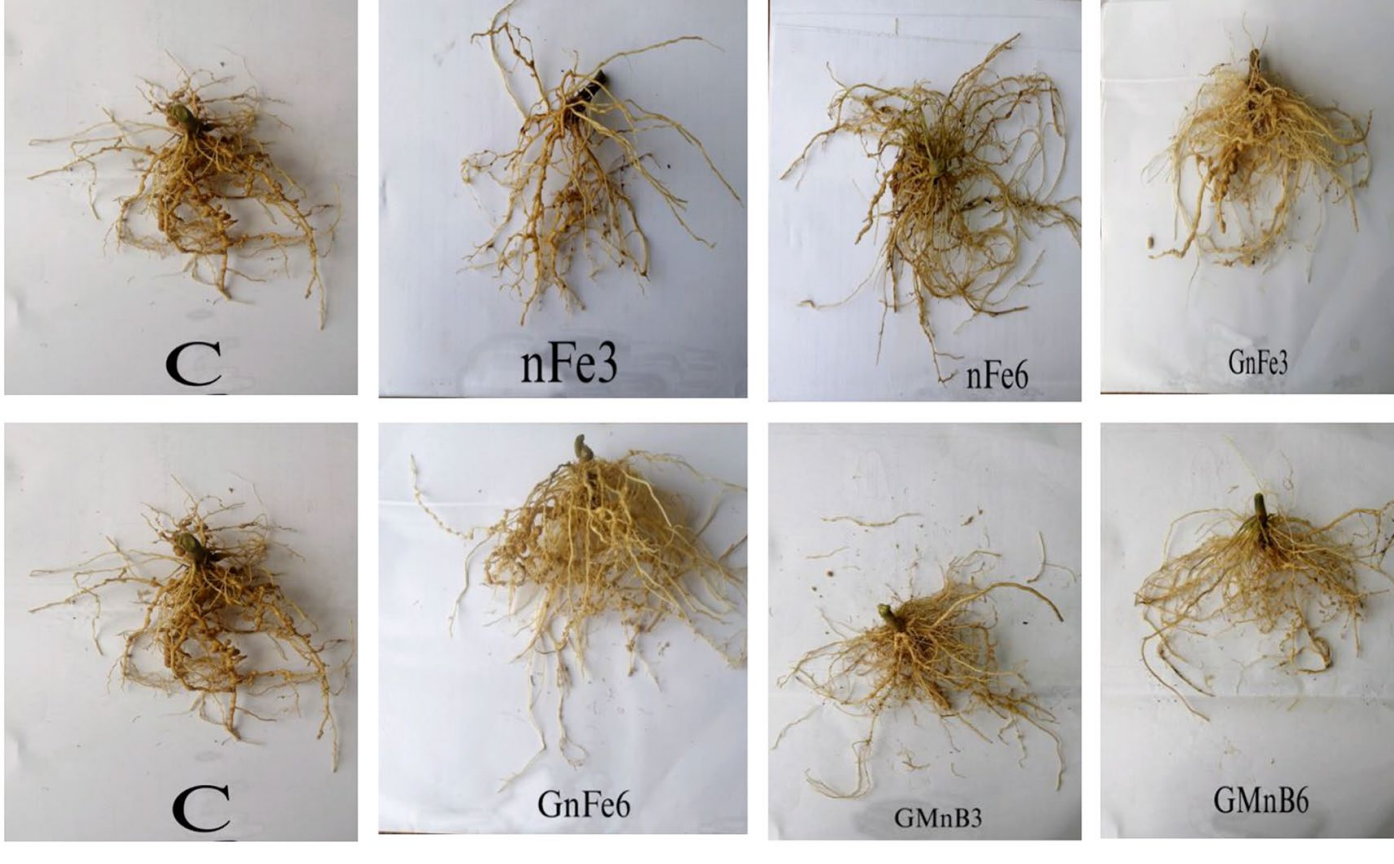
Root gall of nematodes on tomato plant growth during an experiment with iron nanoparticles (nFe), green iron nanoparticles (GnFe), and green magnetic nanobiochar (GMnB).

The total number of root-knot nematodes on tomato plants at varied rates decreased significantly with the addition of the examined nanomaterials (Table 4; Fig. 5). Application of GMnB at 6 mg kg^− 1^ was the most efficient treatment for decreasing tomato plant root-knot nematode populations, with a rise of 66.9% in the number of nematodes per 250 cm of soil, 69.23% in the number of root galls, and 65.18% in the egg masses compared to the untreated soil. The number of root-knot nematode populations decreased as the rates of the nanomaterials studied increased. The addition of GnFe_1_ and GnFe_6_ increased the reduction of root galls of root-knot nematodes by 22.44% and 17.76% compared with nFe_1_ and nFe_2_, respectively. There is no significant difference in the nematode per 250 cm^**3**^ of soil, root galls, or egg masses between the addition of GnFe_3_ and GnFe_6_ treatments.

**Table 4:** The effect of the studied nanomaterials on root-knot nematode populations on the roots of infected tomato plants and in the soil

Treatments	Number of root gall	Number of egg mass	Number of In 250/cm^3^
C	63.08 ^f^	58.33 ^e^	1262 ^d^
nFe_3_	32.17 ^e^	30.33 ^d^	643 ^e^
nFe_6_	28.82 ^d^	28.33 ^b^	576 ^d^
GnFe_3_	24.95 ^b^	29.73 ^b^	550.27 ^c^
GnFe_6_	23.70 ^b^	27.59 ^b^	540.97 ^c^
GMnB_3_	21.62 ^a^	22.59 ^a^	504.34 ^b^
GMnB_6_	19.54 ^a^	20.31 ^a^	427.84 ^a^
F-test	**	**	**
LSD _(0.01)_	1.61	2.33	30.94
LSD _(0.05)_	2.24	3.23	42.94

Where: C: control (NPK recommended doses); nFe_3_: nano iron oxide at 3 mg kg^-1^; nFe_6_ :nano iron oxide at 6 mg kg^-^; GnFe_3_; green nano iron oxide at 3 mg kg^-1^; GnFe_6_: green nano iron oxide at 6 mg kg^-1^; GMnB_3_: green magnetic nanobiochar at 3 mg kg^-1^, and GMnB_6_: green magnetic nanobiochar at 6 mg kg^-1^. Similar letters indicate no significant variations among treatments

### Correlation between egg masses of nematodes or dry weight of tomato plants and total phenolic acids and total flavonoids

**Fig. 6:**
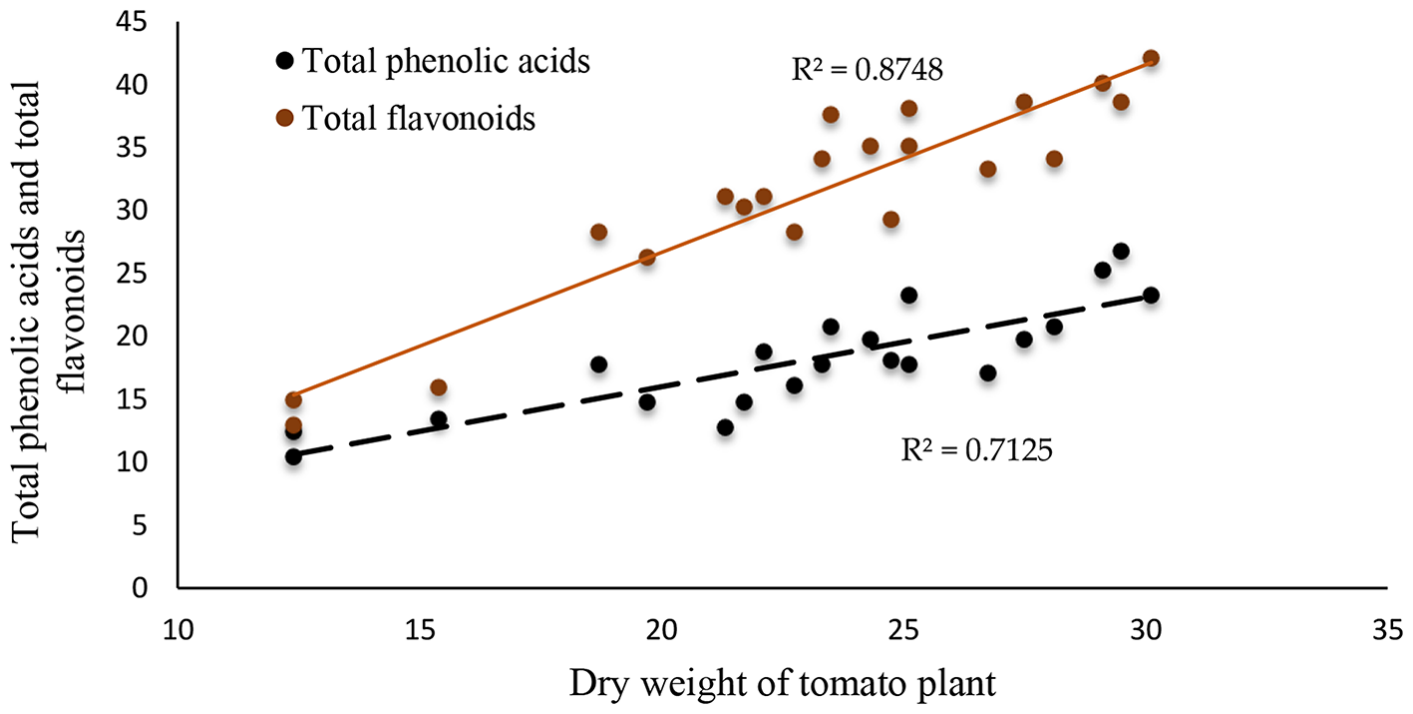
Correlation between dry weight of plant and total phenolic acids and total flavonoids

**Fig. 7:**
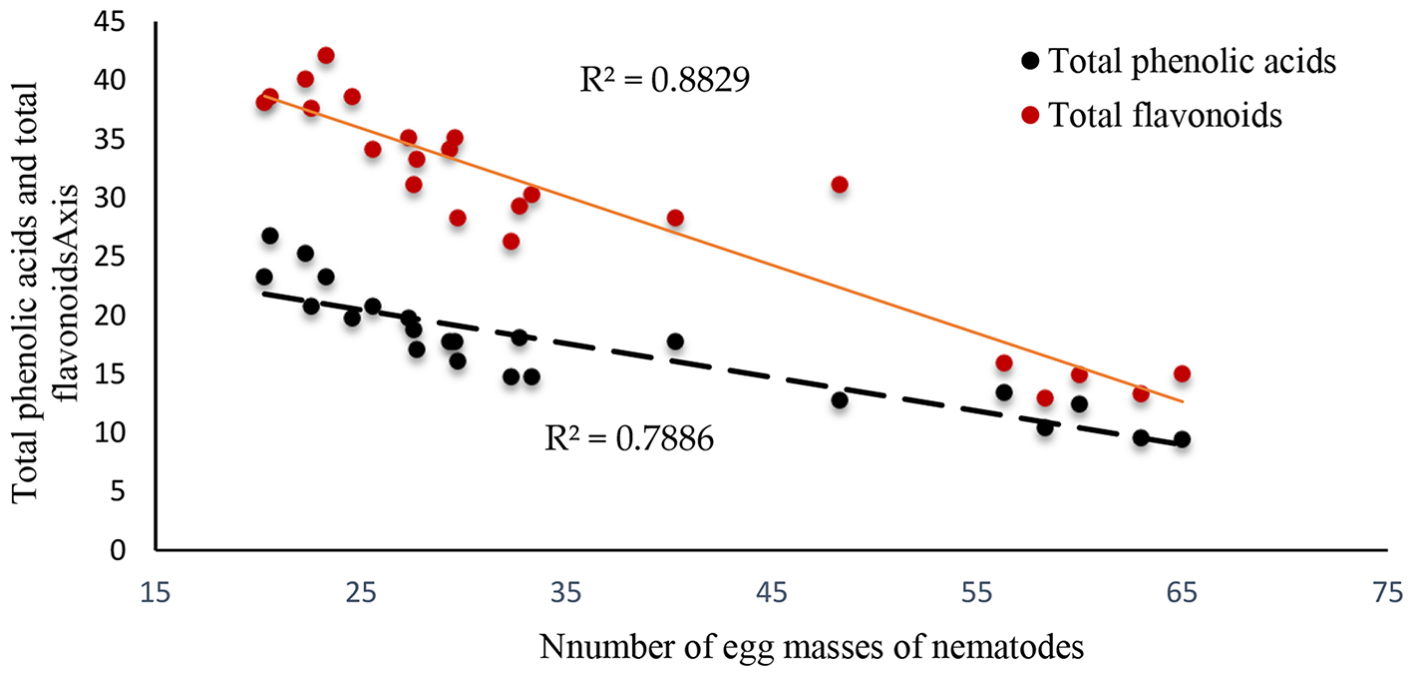
Correlation between number of egg masses of nematodes and total phenolic acids and total flavonoids

Figures 6 and 7 demonstrated the presence of statistically significant correlation coefficients (R^2^) between the number of egg masses of nematodes and total phenolic acids and total flavonoids, which were 0.788 and 0.883, respectively. The correlation coefficients (R^2^) between the dry weight of the tomato plant and total phenolic acids and total flavonoids were 0.7125 and 0.875, respectively.

## Discussion

The bands at 3452.40 cm^− 1^ in GnFe and 3429.85 cm^− 1^ in GMnB were identified as the O-H stretching, which refers to the phenolic group present in the compost tea [39]. In GnFe, the weak band at 3802.21 cm^− 1^ can be attributed to unsaturated nitrogen N-H compounds from compost tea. On the contrary, a sharp band appeared at 3429.85 cm^− 1^ in the GMnB due to the N-H present in the biochar and compost tea together more than that of compost tea alone. The bands at 2088.87 cm^− 1^ in GnFe were indicated by the C-H and H-C = O stretching vibrations [40]. The peaks at 1460.82 cm^− 1^ in GnFe and 1391.85 cm^− 1^ in nFe were identified as the S-H and S = O stretching [41]. This refers to the use of compost tea and ferrous sulfate during the synthesis of green iron oxide nanoparticles. The peaks at 1104 cm^− 1^ in GMnB represent the symmetric C-O and C-N stretching. The peaks at 1639.13 cm^− 1^ in GnFe and 1636.95 cm^− 1^ in GMnB were due to H-O-H and C = C stretching, which indicates phenolic compounds [41]. The Fe-O vibrations spanned between 503.29 and 860.33 cm^− 1^ in all nanomaterials studied, which are consistent with the values obtained by Basavaraja et al. [42]. The presence of organic compounds on the surface of nFe influences its FTIR peaks [43]. The shift in the peaks observed around 537.67 cm^− 1^ in GnFe and 503.29 cm^− 1^ in GMnB instead of the peak of 575.28 cm^− 1^ in nFe may be due to organic molecules from compost tea on the surface of nFe.

The results showed the spinel phase structure of magnetite (Fe_3_O_4_), Fe_3_O_4_TiO, and ferrous sulfate monohydrate, which is consistent with the XRD standard for magnetic iron oxide nanoparticles [24]. Note that the XRD of GnFe and GMnB in the peaks as organic substances, Ca, Al, Mn, and PO_4_; were due to polyphenol groups or other biomolecules containing Ca, Al, Mn, and PO_4_; present in the compost tea. Compost tea contains many beneficial microorganisms, nutrients, humic substances, phytohormones, organic acids, and nutrients [44]. The nFe surfaces are black in color, and the shape of the nanoparticles is spherical with a moderate difference in size. Similar results on TEM analysis of nFe have also been reported by Lida et al. [45]. In this study, the surface area of MGnB was larger than that of nFe and GnFe due to the thermal treatment of biochar at more than 450 °C, which enabled many pores to increase its surface area. Similarly [46], found that the surface area of ball-milled magnetic nanobiochar derived from wheat straw increased with increasing temperature. The high surface area of GMnB and GnFe is important for nutrient uptake by the root and their transport via vascular systems [47].

A wide class of secondary metabolites known as phenolic substances includes phenols, flavonoids, free amino acids, tannins, and their derivatives. Phenolic substances have been identified as antioxidant substances because they have a free hydroxyl group in their structure, which has the ability to scavenge free radicals [48, 49]. Another way to improve tolerance and plant growth in challenging environments is through the formation of phenolic substances [50]. Norouzi et al. [51] found that iron oxide nanoparticles act as abiotic variants in *Dracocephalum kotschyi*, increasing the synthesis of phenolic substances. Following the treatment of iron oxide nanoparticles, elevated phenolic compounds were additionally identified in *Molvadian balm* despite stress from salinity. TPH had a greater concentration in pots that received GnFe and GMnB than in pots supplied with nFe at the identical dose, according to our findings. This occurs because there are many nutrients, humic compounds, and other growth-promoting substances in the compost tea (tea was used for the synthesis). Zhao et al. [52] discovered an increase in protein concentration in the fruit of cucumbers after applying nanoparticles. The increased protein content might be attributed to the upward regulation of enzymes associated with N metabolism as well as the enhanced photosynthetic effectiveness of photosystems I and II [53]. This was supported by a considerable increase in nutritional (N, P, and K) contents in the tomato plants. According to the findings from studies on the effects of AgNPs on potato [54] and Arabidopsis [55], the results demonstrated that raising the level of the studied nanomaterials led to a significant increase in the tomato plant content of total carbohydrate, total free amino acid, flavonoids, and tannins compared to the control treatment. Ashraf et al. [56] observed an increase in photosynthetic pigments, phenolic substances, total flavonoids, and levels of protein in tomato plants after treatment with varied concentrations of nFe. The use of nanoparticles improves biomass levels and chlorophyll content, as well as photosynthetic processes, antioxidant mechanisms, osmolyte production, and carbohydrate content in plant cells. Furthermore, when nanoparticles penetrate plant cells, they not only increase N_2_ levels and protein content, but they also control gene expression during both abiotic and biotic stresses [57, 58]. Application of nano-iron oxide plays an important role in enhancing plant production, protecting tomato plants from root-knot nematodes, and also protecting the host plant from biotic and abiotic stress [59].

All nano-amendments gave higher tomato plant growth and attributes compared to the control (NPK) due to their properties such as high penetration ability, large size, and surface area [60]. In this investigation, nFe additions significantly improved tomato plant growth and attributes. Nanoparticles, according to Govorov and Carmeli [61], can increase plant growth by enhancing the efficacy of chemical energy generation in the photosynthesis process. As the results demonstrated, increasing the amount of pigment produced by sunlight under nFe treatments can promote plant growth and the generation of biomass by improving the process of photosynthesis. According to Mazaherinia et al. [62], the addition of nF improved peanut growth through modulating phytohormone levels and the antioxidant activity of enzymes. Feng et al. [63] concluded that nFe additions to wheat plants can enhance plant growth by improving photosynthetic performance and Fe availability, and P. Shankramma et al. [64] reported that the addition of magnetic nanoparticipants improved tomato plant growth attributes. This increased effect of magnetic nanoparticles could be explained by magnetic iron’s role in promoting N, P, and K uptake, which promotes the growth of plants. By controlling ion transport, eliminating heavy metals, and sustaining root cells, amino acid sequences can accelerate the growth of plants and enhance plant recuperation from stress to their normal biochemistry and osmotic equilibrium [56, 65]. The findings demonstrated that increasing the levels of nano-amendments significantly increased the phenolic substances of the tomato plant compared to the control treatment. Increasing adaptation and enhancing plant growth in challenging environmental conditions can also be accomplished by accumulating phenolic substances [66]. In this study, the decrease in the dry weight of the tomato plant was associated with total phenolic acids (R^2^ = 0.7125) and total flavonoids (R^2^ = 0.875) (Fig. 6). A previous study on Fe nanoparticles by Alam et al. [67] demonstrated that biosynthesized nFe has antibacterial activity against the tomato wilt pathogen *Ralstonia solanacearum* in *vitro* and in *vivo*. This similar application of zinc oxide nanoparticles has antimicrobial activity against bacterial wilt and bacterial leaf spot in tomatoes [68]. Sidorowicz et al. [69] tested Ag NPs synthesized from secondary metabolites extracted from marine algae on *Pseudomonas aeruginosa*, which showed a strong antibacterial effect due to disruption of the outer membrane of *P. aeruginosa*, affecting cell permeability with resulting disturbances called “pits” that lead to cell lysis.

Magnetic iron is distinguished by its strong magnetism and contact with water, resulting in an electromagnetic field that allows beneficial plant nutrients to pass through and eradicate nematodes and bacteria from the rhizosphere of plant roots [70]. Ismail et al. [71] found that the magnetic iron application reduced root-knot nematode numbers in the soil and on the roots of two grape varieties. The nematicidal effect of magnetic nanobiochar can be attributed to its high content of functional groups that interfere with the enzyme-protein structure of nematode cells. It also contains some oxygenated compounds that have lipophilic properties that enable them to dissolve the cytoplasmic membrane of nematode cells. The magnetic biochar nanoparticles are characterized by their small size, high surface area, and magnetic nature, and their field helps them pass the elements useful to agriculture and eliminate nematodes on plant roots [72]. Ohri and Pan [73] found that phenolic compounds protect plants by increasing their resistance against nematode attack. In this work, the decrease in the number of egg masses of nematodes was associated with total phenolic acids (R^2^ = 0.788) and total flavonoids (R^2^ = 0.883) (Fig. 7). Our results showed that the application of green iron oxide nanoparticles and magnetic nanobiochar significantly increased the protein content in tomato plants infected with root-knot nematodes. One defense mechanism is the production of metabolites with anti-nematode activity. Anita et al. [74] found that the accumulation of phenolics and proteins contributed to the control of root knot nematodes. The mechanism of action of flavonoids such as glycine I against nematodes has been elucidated, as they repel J2 juveniles of *M. incognita* and inhibit their respiration. The main mechanism by which green iron nanoparticles (nFe) inhibit root-knot nematode growth is primarily through oxidative stress caused by ROS such as hydrogen peroxide (H_2_O_2_), superoxide radicals (O^− 2^), hydroxyl radicals (-OH), and singlet oxygen (^1^O_2_), which cause protein and DNA damage in root-knot nematodes, ultimately resulting in cell death. And also, induced lipid peroxidation may be one of the mechanisms leading to cell death. Additionally, plants’ defense mechanisms versus nematodes use enzymatic antioxidants [75]. Many studies in the literature on tomatoes demonstrate how the root-knot nematode affects antioxidants, lipid peroxidation, root phenol content, and peroxidase activities as defenses against nematode infection [76, 77].

As a result, treating tomato plants with GnFe or GMnB reduced plant infection by decreasing the number of galls, egg mass, and eggs per egg mass of *M. incognita* in the roots. These results suggest that Fe in the form of green nano plays an important role in the development of plants as a protector against biotic stresses, such as pathogen infection, through the interaction of plant cells with proteins and other biomolecules containing active groups, which enhances the transfer of signals between the living cells and the modification of receptor proteins, which improves a number of plant innate mechanisms in relationship with *M. incognita*.

## Conclusions

The studied nanomaterials were characterized by their high surface area and contained functional groups (such as H-C = O, S-H, S = O, H-O-H, C = C, and Fe-O) and minerals. Its addition improved phenols, flavonoids, free amino acids, total carbohydrate, and tannins in nematode-infected tomato plants and reduced the total number of root-knot nematodes on tomato plants, which had a significant role in promoting tomato growth. GMnB application at 6 mg kg-1 was the most effective treatment to reduce the numbers of root galls, egg mass, and nematodes per 250 cm^3^ of soil compared to other treatments. The decrease in the number of egg masses of nematodes was associated with total phenolic acids (R^2^ = 0.788) and total flavonoids (R^2^ = 0.883). Thus, the results indicate the need to focus on using GMnB at 6 g kg^− 1^ as a natural and environmentally friendly product to reduce nematode numbers, and increase tomato productivity, and thus contribute to sustainable agricultural practices. As a result, it is proposed to identify the genes responsible for reducing the number of nematodes in infected plants as a result of the addition of green nanomaterials.

## Data Availability

The datasets used and analyzed during the current study are available from the corresponding author on reasonable request.
